# PM_2.5_ Concentrations and Subjective Well-Being: Longitudinal Evidence from Aggregated Panel Data from Chinese Provinces

**DOI:** 10.3390/ijerph16071129

**Published:** 2019-03-29

**Authors:** Pan Zhang, Zhiguo Wang

**Affiliations:** 1 School of International and Public Affairs, Shanghai Jiao Tong University, Shanghai 200030, China; 2 China Institute for Urban Governance, Shanghai Jiao Tong University, Shanghai 200030, China; 3 School of Engineering, The Hong Kong University of Science and Technology, Hong Kong 999077, China; brainw@ust.hk; 4 Business School, The Hong Kong University of Science and Technology, Hong Kong 999077, China

**Keywords:** level of happiness, PM_2.5_, inequality of happiness, environmental governance

## Abstract

Although haze pollution with PM_2.5_ as the chief pollutant has become a critical threat worldwide, little research has examined the effects of PM_2.5_ concentrations on subjective well-being. Based on a longitudinal aggregated panel dataset from Chinese provinces, this study investigates the effects of PM_2.5_ concentrations on levels of happiness and the inequality of happiness. The results showed that high ground-level PM_2.5_ concentrations decreased the average level of happiness and high PM_2.5_ concentrations had stronger negative effects on the happiness of persons with high income than those with low income. In addition, PM_2.5_ concentrations were also significantly negatively related to inequality of happiness in Chinese provinces. Further empirical tests showed that the negative effects of PM_2.5_ concentrations on the inequality of happiness could be explained by the stronger influence of PM_2.5_ concentrations on the subjective well-being of individuals with a higher initial level of happiness than those with a lower initial level of happiness. This confirms that PM_2.5_ pollution can do harm to subjective well-being and reduce variations in the subjective well-being of individuals. The policy implications of controlling haze pollution and improving well-being are discussed.

## 1. Introduction

Improving subjective well-being is one of the most important missions of governments [1]. Subjective well-being refers to one’s overall perception about her/his life [2] and it is usually measured by two interchangeable items: happiness and life satisfaction [3]. Previous research has generally explored the influences of micro factors on the subjective well-being of individuals, including demographic factors and the socio-economic status of individuals [3]. Recently, macro factors have attracted scholars’ attention due to their policy importance, such as the quality of government [1,4,5] and economic conditions [6,7,8]. Particularly, air pollution is becoming a prominent problem in the world [9,10] and many studies have confirmed that air pollution does great harm to human’s physical health [11,12], environmental economic scientists have definitely emphasized the urgency of evaluating the effects of pollution on subjective well-being [13,14].

Recently, studies examining the relationship between environmental pollution and subjective well-being have emerged, but there are still at least the following two critical research gaps. First, current studies have examined the effects of some air pollutants on subjective well-being, including PM_10_ [15,16,17,18,19], sulfur dioxide [20,21,22,23,24,25], nitrogen dioxide [19,20,25,26,27,28], and carbon emissions [16,29]. Actually, PM_2.5_ is the chief pollutant in China’s emerging haze pollution [30] and it is most harmful to human’s physical health among pollutants [31]. However, little research has examined the relationship between PM_2.5_ concentrations and subjective well-being. Second, the level of happiness and the variations in happiness (or the inequality of happiness) have been two critical topics in subjective well-being research [7,32]. Nevertheless, the current literature has mainly analyzed the effects of air pollution on the level of subjective well-being [3,13], with no research examining the effects of air pollution on the inequality of subjective well-being.

Against this background, this work constructed a unique panel dataset from Chinese provinces, including the annual PM_2.5_ concentrations, average happiness levels, and happiness inequality. Running a set of panel data models, it aims at investigating the effects of annual PM_2.5_ concentrations on the average level of happiness and the inequality of happiness across Chinese provinces. Its theoretical contributions are two-fold. On the one hand, this study is the first to examine the impacts of PM_2.5_ concentrations on subjective well-being. In view that PM_2.5_ is the chief pollutant in haze pollution and little research pays attention to how PM_2.5_ concentrations influence subjective well-being, it provides new evidence about the well-being loss effects of PM_2.5_ pollution. On the other hand, previous studies have just focused on the effects of air pollution on individuals’ levels of happiness [3,13], while this study takes the first step to investigate the effects of air pollution on the inequality of happiness by focusing on PM_2.5_. Therefore, this study contributes to the current literature by uncovering the effects of annual ground-level PM_2.5_ concentrations on variations of individuals’ subjective well-being.

## 2. Literature Review

Examining the effects of air pollution on people’s subjective well-being is an important research issue in environmental economics [3]. Due to the difficulty of directly measuring the monetary value of air quality, economists have developed a novel approach to assessing air quality using happiness data, called the “happiness approach” [13]. This approach considers people’s subjective well-being to be a function of air pollution, income, and other factors [13]. In general, it assumes that income is positively related to subjective well-being, while air pollution is negatively related to subjective well-being. Then, to keep subjective well-being stable, the substitution relationship between air quality and income is calculated based on the marginal effects of air pollution and income on subjective well-being [13,33]. Although some studies have quantified the equivalent monetary value of air quality based on subjective well-being data, not all types of air pollution have significant negative effects on subjective well-being [26,29].

Overall, the literature on the effects of air pollution on subjective well-being can be divided into two clusters. The first cluster examined how people’s perceptions of the severity of air pollution influenced their subjective well-being [34,35,36,37,38]. For example, perceived risks related to the intensity of exposure to polluted air and the hazards of pollutants have been found to be significantly negatively related to respondents’ happiness in Jinchuan, China [36]. In addition, some scholars have confirmed the significant negative effects of perceived air pollution and noise on life satisfaction in Germany [35]. However, Wang and Cheng [38] found that the perceived severity of environmental issues had little effect on happiness in China. Interestingly, some scholars provided evidence that endogenous perceived air pollution was negatively related to happiness in Germany [37]. Moreover, when objective air pollution was used as the instrumental variable, the negative effect of perceived air pollution was not significant [37].

The second cluster used objective air pollution indicators to investigate the effects of air pollution on people’s subjective well-being. Specifically, most studies have examined the correlations between subjective well-being and air pollution, as measured by annual pollutant concentrations [16,17,18,20,22,24,27,28]. For example, Welsch [26] found that nitrogen dioxide was negatively related to subjective well-being in European countries. Menz [15] provided cross-national evidence of the negative relationship between PM_10_ and life satisfaction. Finally, Luechinger [23] revealed that annual mean sulfur dioxide concentrations were negatively related to life satisfaction in 13 European countries. These studies proved the effects of exposure to air pollution on subjective well-being in the medium and long term, but failed to reveal the short-term effects of air pollution on subjective well-being. As a result, some studies have begun to fill this gap by matching self-reported well-being data with daily air quality data when respondents are surveyed [29,33,39]. For example, Levinson [28] used matched daily data to confirm that daily PM_10_ pollution was significantly related to happiness in the U.S. Using a similar method, Zhang, Zhang, and Chen [39] found that a high air pollution index was negatively related to hedonic happiness and positively related to depression, but had little impact on life satisfaction in China.

Although some studies have contributed to the current literature by investigating the relationship between air pollution and subjective well-being, there are still some limitations. First, current studies have mainly focused on the effects of air pollution index or air quality index [39,40], PM_10_ [15,16,17,18,19], sulfur dioxide [20,21,22,23,24,25], nitrogen dioxide [19,20,25,26,27,28], and carbon emissions [16,29]. However, PM_2.5_ is the dominating pollutant of haze pollution in most parts of China and is known to pose a greater threat to human health than PM_10_ [9]. Yet, to the best of our knowledge, only several studies have empirically examined the effects of PM_2.5_ on subjective well-being recently [29,31,40]. For example, investigating the effects of six pollutants on hedonic happiness using well-matched daily data in China, including PM_2.5_, PM_10_, nitrogen dioxide, carbon monoxide, ozone, and sulfur dioxide, Zhang, Zhang, and Chen [29] showed that only PM_2.5_ and PM_10_ significantly decreased hedonic happiness.

Second, current research has mainly examined the effects of air pollution on the level of subjective well-being, which corresponds to the level of happiness (or life satisfaction) of each individual or the average level of happiness (or life satisfaction) of respondents in each region [3,13]. However, the distribution of subjective well-being in the population, as measured by the inequality of subjective well-being, has been another critical topic in well-being research [32]. Yet no research has examined the relationship between air pollution and the inequality of subjective well-being. Third, current evidence of the relationship between air pollution and subjective well-being has mainly come from developed countries or regions, including European regions [23,24,26], the U.S. [33], the UK [28], Germany [22], Australia [17], and Spain [16]. As developing countries are increasingly affected by severe air pollution, especially China [9,30], further research is needed to revisit the effects of air pollution on subjective well-being in developing countries.

## 3. Methodology

### 3.1. Model Specifications

This study constructed a panel dataset of 25 mainland Chinese provinces from 2003 to 2015 to examine the effects of annual PM_2.5_ concentrations on the average level of happiness and the inequality of happiness. Nine provincial jurisdictions in China were excluded from the analysis because of the unavailability of happiness data, namely Inner Mongolia, Hainan, Tibet, Qinghai, Ningxia, Xinjiang, Hong Kong, Macao, and Taiwan. The provinces in the sample are located in eastern, western, and central China. Thus, based on a nationwide sample in China, this study complements previous cross-national studies [15,20,23,26,27] and those conducted in a certain sub-region of a country [17,28,36]. In addition, focusing on the Chinese provincial level has the advantage of maximizing variations in the level of happiness and the inequality of happiness between regions, thus providing a solid basis for examining the effects of PM_2.5_ concentrations. The empirical model used was the following:

H_i,t_ = α + β × PM_i,t_ + γ × C_i,t−1_ + ε_i,t_, (1)

where H_i,t_ is the dependent variable, denoting the average level of happiness or the inequality of happiness of each sample province in the year under study, PM_i,t_ is the key explanatory variable and the annual PM_2.5_ concentrations in each province, C_i,t−1_ is a vector composed of all of the control variables, which were derived from the literature on determinants of subjective well-being, ε_i,t_ is the error term, and α, β, and γ are the regression coefficients. All of the control variables were lagged for one year to avoid possible reverse causal relationships, while the PM_2.5_ concentration variable was not lagged due to the timely effect of haze pollution.

Based on Equation (1), we first ran a panel data model to examine the impacts of PM_2.5_ concentrations on the average level of happiness using all the samples. Then, we divided the sample Chinese provinces into two groups according to the urban disposable income per capita: the high income group and the low income group. By examining the effects of PM_2.5_ concentrations on the average level of happiness in these two groups respectively, we identified the heterogenous effects of PM_2.5_ concentrations. In addition, we also ran a panel data model to investigate the relationship between PM_2.5_ concentrations and the inequality of happiness. The influences of PM_2.5_ concentrations on the inequality of happiness proved statistically significant, and then, we also divided the sample Chinese provinces into two groups according to the initial average level of happiness: the high happiness group and the low happiness group. By examining the effects of PM_2.5_ concentrations on the level of happiness in the high happiness group and the low happiness group respectively, we explained the mechanism behind the negative influences of PM_2.5_ concentrations on the inequality of happiness. Particularly, when we ran each panel data model, the Hausman test was conducted to choose the appropriate model between the fixed-effects model and the random-effects model.

### 3.2. Dependent Variables

To construct a longitudinal panel dataset, we aggregated happiness data from all surveyed individuals located in the same province from the same wave of the Chinese General Social Survey (CGSS). The CGSS is a representative nationwide survey in China with a multi-stage stratified probability proportional to size sampling method [41]. The CGSS was conducted for 10 waves, in 2003, 2004, 2005, 2006, 2008, 2010, 2011, 2012, 2013, and 2015. Nine waves of data were used in this study (2004 was excluded due to data unavailability). The CGSS is one of the most used data sources in academic research on Chinese issues because of its high quality, large sample, long series, and broad representation [38,41].

Happiness data came from the following survey question: “In general, to what extent do you think your life is happy?” The respondents were asked to choose one of five answers: 1 (very unhappy), 2 (unhappy), 3 (just so so), 4 (happy), and 5 (very happy). All waves of the CGSS used the same five-level Likert scale to collect information on respondents’ self-reported happiness to obtain comparable happiness data across waves. To measure the average level of happiness, we calculated the mean values of self-reported happiness levels for all respondents in the same province for each wave of CGSS. Inequality of happiness was measured by the standard deviations of self-reported happiness levels for all respondents in the same province for each wave of CGSS. Both measures have been used in previous studies published in high-ranking, peer-reviewed quality of life journals [5,7].

### 3.3. Independent Variables

The independent variable was annual PM_2.5_ concentrations in the Chinese provinces under study. In general, obtaining high quality data on longitudinal PM_2.5_ concentrations is a big challenge for empirical studies investigating haze pollution issues in China, as the first PM_2.5_ monitoring stations and official PM_2.5_ data only appeared in 2013 [9]. To obtain accurate PM_2.5_ concentration data in the Chinese provinces, we retrieved the PM_2.5_ concentration data from each grid from the global annual PM_2.5_ grids developed by a famous international research team [42,43]. By combining aerosol optical depth retrievals from multiple satellite instruments, this approach made it possible to estimate annual ground-level PM_2.5_ concentrations (μg/m^3^) from 1998 to 2016, after removing dust and sea salt. Based on these gridded datasets, we agglomerated them to obtain annual ground-level PM_2.5_ concentrations for each Chinese province for each year.

### 3.4. Control Variables

To reduce omitted variable bias, we controlled a set of factors that could affect subjective well-being. First, previous studies at the individual level have discussed the effects of individuals’ demographic variables on subjective well-being (e.g., age, gender, education, income, employment status) [44]. Therefore, we controlled the proportion of older people, the sex ratio, the ratio of people with a Bachelor’s degree or above to the total population, disposable income per capita, and the unemployment rate. Second, previous studies confirmed that health status was an important predictor of happiness [44]. Because not all waves of the CGSS collected data about respondents’ self-reported health status, we used the outpatient service frequency per capita in each province as a proxy to control the physical health status of persons. Third, some studies at the regional level have argued that certain macro conditions can affect subjective well-being, especially inflation [6], urbanization [45], and income inequality [8,46]. Thus, we controlled the consumer price index, the urbanization rate in each province, the Atkinson Index estimated by previous research to reflect income inequality [47], and the total population. The detailed measures of the dependent, independent, and control variables are presented in Table 1, including their descriptive statistics.

## 4. Empirical Results

**Figure 1:**
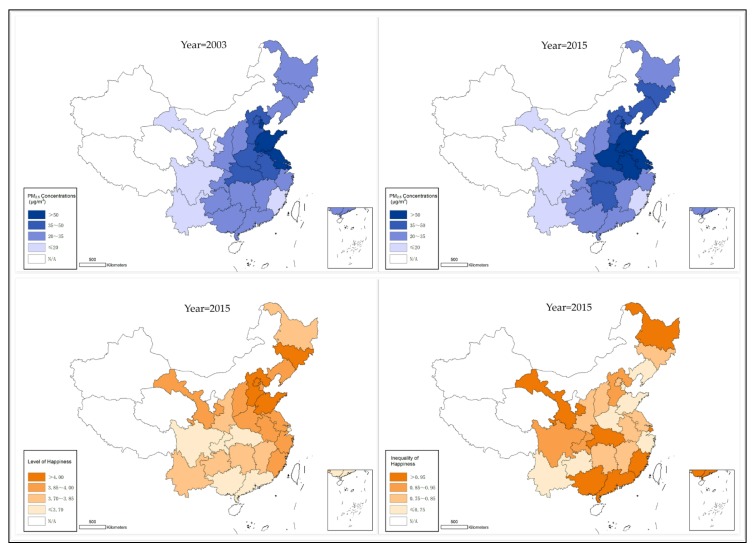
Spatial distribution of PM_2.5_ concentrations, the level of happiness, and inequality of happiness in selected years.

Figure 1 shows the spatial distribution of annual ground-level PM_2.5_ concentrations, the average level of happiness, and the inequality of happiness in selected years. Annual PM_2.5_ concentrations showed large variations between Chinese provinces. For example, some provinces suffered from more severe haze pollution, such as Tianjin, Shandong Jiangsu, and Shanghai. However, PM_2.5_ concentrations in other provinces, especially Yunnan, Sichuan, Fujian, and Gansu, were lower. Besides, the average level of happiness in some provinces was higher in 2015 (e.g., Beijing, Shandong, and Jilin), while the average level of happiness in some other provinces was lower in that year (e.g., Guangxi, Sichuan, Guangdong, and Hubei). In addition, the inequality of happiness in Chinese provinces seemed also significantly different. The inequality of happiness in Guangdong, Gansu, Fujian, and Shanghai was higher than that in other provinces, but the inequality of happiness in Shandong, Henan, and Guizhou was lower.

**Figure 2:**
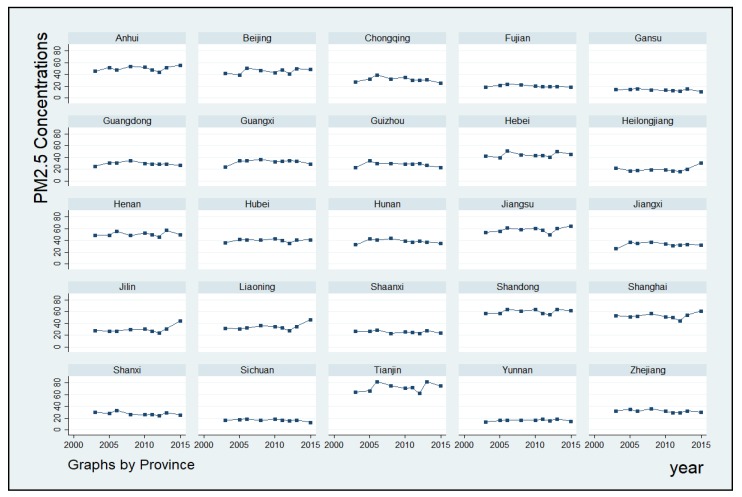
Annual Average Ground-level PM_2.5_ Concentrations in the Chinese Provinces.

Figure 2 presents the temporal trends of annual ground-level PM_2.5_ concentrations in Chinese provinces. As this study used annual ground-level PM_2.5_ concentrations without dust and sea salt, the PM_2.5_ concentrations obtained were lower than those reported in previous research [9,30]. Overall, despite strong fluctuations, mean of the annual average PM_2.5_ concentrations increased gradually over time. According to our calculation, mean of the annual average PM_2.5_ concentrations in Chinese provinces increased from 33.57 μg/m^3^ in 2003 to 37.40 μg/m^3^ in 2015. In addition, the annual ground-level PM_2.5_ concentrations in some provinces increased more significantly over time, such as Heilongjiang, Jilin, Liaoning, Anhui, and Jiangsu. Nevertheless, PM_2.5_ concentrations in some other provinces remained a little more stable.

**Figure 3:**
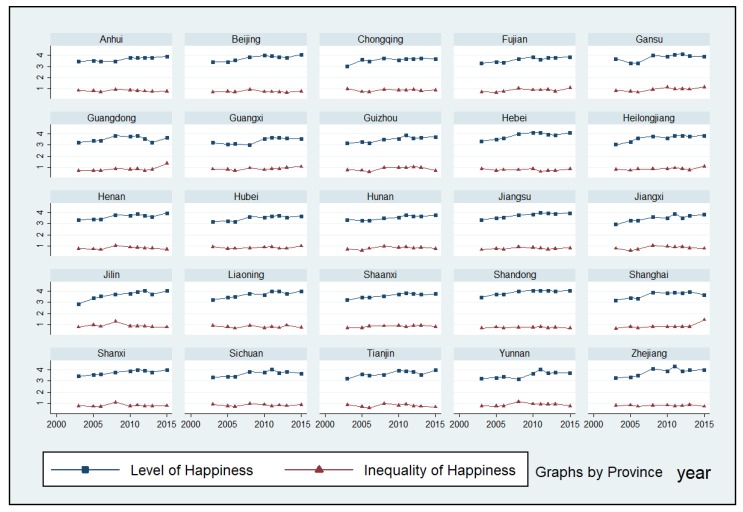
The level of Happiness and Inequality of Happiness in the Chinese Provinces.

In addition, Figure 3 shows the level of happiness and the inequality of happiness by province over the nine waves of the CGSS. It illustrates the significant increase in the average level of happiness in most Chinese provinces. Indeed, the average level of happiness in most provinces increased from about three in the first wave of the CGSS to about four in the last wave. This indicates that the happiness of most Chinese people increased over the last decade. However, the inequality of happiness in the Chinese provinces showed a weaker upward trend. According to our calculation, the mean happiness inequality in the Chinese provinces during the first wave of the CGSS was 0.7985, while it was 0.885 during the last wave. This suggests a general increase in variations in individuals’ self-reported happiness in the provinces. Moreover, this implies that the increase in the level of happiness was also accompanied by an increase in the inequality of happiness in the Chinese provinces.

In Table 2, Model 1 presents the estimated effects of PM_2.5_ concentrations on the average level of happiness using all the samples. In Model 1, the coefficient of PM_2.5_ was −0.006 and statistically significant (*p* < 0.05), supporting the negative effects of high PM_2.5_ concentrations on individuals’ subjective well-being. Specifically, when annual ground-level PM_2.5_ concentrations increased by one μg/m^3^, the average level of happiness decreased by 0.006. Therefore, reducing PM_2.5_ concentrations may be a prospective policy measure to increase subjective well-being. Interestingly, among the control variables, Income level was significantly positively related to the average level of happiness (1.666, *p* < 0.01). Then, Models 2 and 3 present the estimated impacts of PM_2.5_ concentrations on the average level of happiness using the samples in the high income group and those in the low income group, respectively. PM_2.5_ concentrations were significantly negatively related to the average level of happiness in the high income group (Model 2, −0.0051, *p* < 0.1), while the coefficient of PM_2.5_ was −0.0047 and not significant (Model 3, *p* > 0.1) in the low income group. This means PM_2.5_ pollution had greater negative effects on the subjective well-being of individuals with high income than those with low income.

In addition, Model 4 in Table 3 shows the estimated impacts of PM_2.5_ concentrations on the inequality of happiness based on all the samples. The coefficient of PM_2.5_ was −0.0012 and was statistically significant (*p* < 0.05) in Model 4, confirming that high PM_2.5_ concentrations reduced the inequality of happiness. Specifically, the standard deviation of individuals’ self-reported happiness decreased by 0.0012 when annual ground-level PM_2.5_ concentrations increased by 1 μg/m^3^. One explanation may be that PM_2.5_ pollution has a stronger negative effect on the happiness of individuals with a higher initial level of happiness than those with a lower initial level of happiness, reducing the happiness gap between these two groups of people. Regression results of Models 5 and 6 confirmed this argument. Specifically, the regression coefficients of PM_2.5_ on the level of happiness in the high happiness group (see Model 5) and in the low happiness group (see Model 6) were both significantly negative (−0.0081, *p* < 0.05; −0.0062, *p* < 0.1), while the absolute value of the coefficient of PM_2.5_ in the high happiness group was greater than the absolute value of the coefficient of PM_2.5_ in the low happiness group.

Most control variables had inconsistent effects on the level of happiness and the inequality of happiness according to Models 1 and 4. For example, Income level and Income inequality had strongly significant effects on the average level of happiness in Model 1, while only Population and Income level had significant effects on the inequality of happiness in Model 4. This implies that the determinants of the level of happiness and the inequality of happiness may be significantly different. Therefore, future research should pay more attention to the level of happiness and the inequality of happiness simultaneously. It is worth noting that Income level was significantly positively related to the average level of happiness (Model 1, 1.666, *p* < 0.01) and the inequality of happiness (Model 4, 0.232, *p* < 0.01). This strongly supports that income played an important role in individuals’ subjective well-being. Surprisingly, the coefficients of Outpatient were not significant in Models 1 and 4. A possible explanation may be the limitation that the outpatient service frequency per capita as a proxy could not reflect the health status of respondents directly and accurately.

## 5. Discussion

High PM_2.5_ concentrations turned out to be significantly negatively related to the level of happiness in this study. We can make two possible explanations: the direct mechanism and the indirect mechanism, which are similar to the mechanisms behind negative effects of environmental pollution discussed in previous studies [18]. The first reason is that PM_2.5_, the chief pollutant in haze pollution, may directly affect subjective well-being. Previous studies have shown that PM_2.5_ can affect individuals’ emotions, including increasing depression [48] and negative emotions [49]. Thus, PM_2.5_ pollution is also expected to directly affect individuals’ judgments of their quality of life [18]. Specifically, high PM_2.5_ concentrations or severe haze pollution can mainly result in low or limited visibility. Then, low visibility in haze days can easily increase individuals’ negative emotions and decrease their positive feelings about their quality of life.

The second reason is that high PM_2.5_ concentrations may also affect subjective well-being indirectly. First, PM_2.5_ is expected to indirectly reduce subjective well-being by affecting physical health. For example, previous studies have found that PM_2.5_ pollution poses a significant risk to physical health [11] and then, poor physical health is usually negatively related to subjective well-being [44]. Second, PM_2.5_ concentrations could hinder economic growth [50] and low wealth would reduce subjective well-being [44]. Thus, PM_2.5_ can also indirectly do harm to subjective well-being by hindering economic development. Third, previous studies also confirmed that, when PM_2.5_ concentrations were high, individuals tended to engage in less physical activities and visits to the outdoors, which would then reduce their subjective well-being [31]. All these three channels above provide an incomplete picture about how PM_2.5_ concentrations may influence subjective well-being indirectly.

In addition, the findings show that PM_2.5_ concentrations had a significant negative relationship with the inequality of happiness, which means PM_2.5_ concentrations reduced variations in the subjective well-being of individuals in each province. Previous studies mainly concentrate on the relationship between air pollution and the average level of subjective well-being [3,13], but no research examines the impacts of PM_2.5_ concentrations on the variations in subjective well-being in the population. This research gap hinders our systematic understanding of the “pollution—subjective well-being” nexus. This work takes the first step to examine how air pollution influences the inequality of happiness by focusing on PM_2.5_. Thus, it adds new knowledge in this field by uncovering the effects of annual ground-level PM_2.5_ concentrations on the inequality of individuals’ subjective well-being in China.

The negative relationship between PM_2.5_ concentrations and the inequality of happiness can be understood as follows. Indeed, PM_2.5_ concentrations can reduce individuals’ subjective well-being [29], while the negative effects of PM_2.5_ concentrations on individuals’ happiness are expected to be heterogeneous. PM_2.5_ pollution is expected to have a greater effect on individuals with a higher initial level of happiness than those with a lower level of happiness. This argument was confirmed by the regression results by using sub-samples in the high happiness group (see Model 5) and those in the low happiness group (see Model 6). In general, people with a higher level of happiness are those with higher income, better education background, and social status. Because they are habituated to a high quality of life, the subjective well-being of these individuals is expected to be more vulnerable to external shocks. When their lives are affected by severe haze pollution, they are more likely to judge that the decrease of quality of life is more dramatic. Thus, the heterogeneous effects of PM_2.5_ concentrations on the subjective well-being of these two groups of individuals would promote the equality of happiness.

## 6. Conclusions

In the context of China’s severe haze pollution with PM_2.5_ as the chief pollutant [9,30], this study examined the well-being effects of PM_2.5_ concentrations in the Chinese provinces. To this end, a unique panel dataset from Chinese provinces was constructed, including annual ground-level PM_2.5_ concentrations, average happiness level, and happiness inequality. Panel data analysis techniques were used to conduct the empirical analysis. The results showed that high annual ground-level PM_2.5_ concentrations had significant negative effects on the average level of happiness. Specifically, when annual ground-level PM_2.5_ concentrations increased by 1 μg/m^3^, the average level of happiness decreased by 0.006. Besides, PM_2.5_ concentrations showed stronger negative effects on the happiness of individuals with high income than those with low income. In addition, high annual ground-level PM_2.5_ concentrations also reduced the inequality of happiness and the standard deviation of individuals’ self-reported happiness decreased by 0.0012 when annual PM_2.5_ concentrations increased by 1 μg/m^3^. Further empirical tests confirmed that the negative effects of PM_2.5_ concentrations on the inequality of happiness resulted from the stronger influence of PM_2.5_ concentrations on the subjective well-being of individuals with a higher initial level of happiness than those with a lower initial level of happiness.

The findings of this study have important policy implications. First, the study confirms that high PM_2.5_ concentrations can do harm to subjective well-being, and environmental economic literature usually assesses the monetary value of air quality with the “happiness approach” [13]. As mentioned earlier, previous studies have found that PM_2.5_ pollution can hinder economic growth [50] and threaten people’s physical health [11]. Thus, it is necessary to control PM_2.5_ concentrations, especially setting mandatory performance targets [30,51], improving energy utilization efficiency [52,53], developing renewable energies [54,55] and updating the economic structure [9]. However, to control haze pollution needs high fiscal expenditure or even to slow down economic growth. Thus, when conducting a cost-benefit analysis for haze pollution control, all benefits should be calculated, including the increase in subjective well-being and the reduction of diseases. Second, high PM_2.5_ concentrations can reduce the inequality of happiness. In other words, to control haze pollution may increase the inequality of happiness. However, the inequality of well-being is a major threat to social stability. Therefore, our findings emphasize that policy measures should be adopted to decrease the inequality of happiness when efforts are devoted to controlling PM_2.5_ concentrations.

Although it is the first to investigate the effects of PM_2.5_ concentrations on the average level of happiness and the inequality of happiness systematically, this study has some limitations, which leave room for future research. First, this study was conducted in the Chinese context. However, some global data sources on subjective well-being are available, containing data from most countries, such as the World Values Survey. Therefore, future studies should examine the effects of PM_2.5_ concentrations on subjective well-being using a cross-national analysis. Second, this study investigated the correlation relationship between PM_2.5_ concentrations and subjective well-being. However, experimental or quasi-experimental designs have become increasingly popular for examining causal relationships [55]. Thus, using an experimental design offers a prospective avenue to investigate the causal relationship between PM_2.5_ concentrations and subjective well-being.

## Figures and Tables

**Table 1:** Measures, data sources, and descriptive statistics.

Variables	Measures	Sources	Mean	S.D.
Level of happiness	The mean value of respondents’ self-reported happiness in each Chinese province in the investigated year	CGSS	3.65	0.28
Inequality of happiness	The standard deviation of respondents’ self-reported happiness in each Chinese province in the investigated year	CGSS	0.83	0.12
PM_2.5_	Annual average PM_2.5_ concentrations in each Chinese province in the investigated year (μg/m^3^)	[42,43]	36.21	15.13
Unemployment	The registered urban unemployment rate of each Chinese province in the year before the investigated year	CSY	3.64	0.72
Gender	The male-to-female sex ratio of each Chinese province in the year before the investigated year	CSY	104.03	3.51
CPI	The consumer price index of each Chinese province in the year before the investigated year	CSY	102.49	2.13
Elder	The number of people aged 65 and over divided by the total population in each Chinese province in the year before the investigated year	CSY	9.32	1.62
Education	The number of people with a Bachelor’s degree or above divided by the total population in the year before the investigated year	CSY	9.09	6.28
Population	The base-10 logarithm of the total population of each Chinese province in the year before the investigated year	CSY	3.64	0.23
Income level	The base-10 logarithm of urban disposable income per capita in each Chinese province in the year before the investigated year	CSY	4.18	0.21
Urbanization	The urban population divided by the total population in each Chinese province in the year before the investigated year	CSY	50.54	15.87
Income inequality	The Atkinson Index used to reflect income inequality in each Chinese province in the year before the investigated year	[47]	0.342	0.062
Outpatient	The outpatient service frequency per capita in each Chinese province in the year before the investigated year	CSY; CHSY	1.619	1.135

Notes: CGSS refers to the Chinese General Social Survey; CSY refers to China Statistical Yearbooks; CHSY refers to China Health Statistics Yearbooks.

**Table 2:** Impacts of PM_2.5_ concentrations on the level of happiness and their heterogenous effects.

	Model 1		Model 2		Model 3	
	All the Samples		High Income Group		Low Income Group	
	Coef.	S.D.	Coef.	S.D.	Coef.	S.D.
PM_2.5_	−0.006 **	(0.002)	−0.0051 *	(0.00248)	−0.0047	(0.0034)
Unemployment	−0.0287	(0.043)	−0.154	(0.0873)	0.0118	(0.0307)
Gender	−0.0012	(0.004)	−0.0012	(0.0052)	0.0028	(0.0062)
CPI	0.0044	(0.0056)	−0.0053	(0.0076)	0.0074	(0.0062)
Elder	0.0119	(0.0153)	0.0149	(0.0178)	0.0132	(0.0299)
Education	−0.0061	(0.0072)	−0.0014	(0.0106)	−0.0075	(0.0117)
Population	1.074 *	(0.573)	1.532 *	(0.828)	−0.602	(1.393)
Income level	1.666 ***	(0.246)	1.371 ***	(0.342)	2.096 ***	(0.300)
Urbanization	−0.0116	(0.0079)	−0.0012	(0.009)	−0.0155 **	(0.0061)
Income inequality	2.557 ***	(0.725)	3.674 **	(1.204)	0.202	(1.728)
Outpatient	−0.0634	(0.0695)	−0.0735	(0.069)	−0.436	(0.308)
Constant	−7.468 ***	(2.181)	−7.426 **	(3.099)	−2.679	(4.810)
N	225		117		108	
R^2^	0.7059		0.7048		0.7453	
Hausman Test	42.09 ***		36.42 ***		29.61 ***	
Model	Fixed-effects		Fixed-effects		Fixed-effects	

Notes: *** *p* < 0.01, ** *p* < 0.05, * *p* < 0.1. Robust standard errors are shown in parentheses. Within R^2^ is reported for the fixed-effects models.

**Table 3:** Impacts of PM_2.5_ concentrations on the inequality of happiness and the mechanism.

	Model 4		Model 5		Model 6	
	DV: Inequality of Happiness		DV: Level of Happiness		DV: Level of Happiness	
	All the Samples		High Happiness Group		Low Happiness Group	
	Coef.	S.D.	Coef.	S.D.	Coef.	S.D.
PM_2.5_	−0.0012 **	(0.0006)	−0.0081 **	(0.0035)	−0.0062 *	(0.0033)
Unemployment	0.0191	(0.0182)	−0.092	(0.0901)	0.0188	(0.0383)
Gender	0.0042	(0.0026)	0.0015	(0.0058)	−0.0039	(0.0076)
CPI	0.0043	(0.0031)	−0.0021	(0.0062)	0.0079	(0.0084)
Elder	−0.0015	(0.0058)	−0.0173	(0.0257)	0.0273	(0.0157)
Education	−0.0042	(0.0041)	−0.0109	(0.0107)	−0.0162	(0.0118)
Population	−0.121 ***	(0.0336)	1.975	(1.247)	2.176 **	(0.87)
Income level	0.232 ***	(0.0596)	1.040 **	(0.402)	2.141 ***	(0.18)
Urbanization	−0.0007	(0.0018)	0.0075	(0.0112)	−0.0139 **	(0.006)
Income inequality	0.136	(0.247)	3.406 ***	(1.025)	2.577 ***	(0.671)
Outpatient	0.0058	(0.0318)	0.0025	(0.06)	−0.193 ***	(0.0601)
Constant	−0.569	(0.414)	−8.560	(4.780)	−13.30 ***	(3.601)
N	225		108		117	
R^2^	0.1707		0.7328		0.7356	
Hausman Test	15.13		35.04 ***		30.67 ***	
Model	Random-effects		Fixed-effects		Fixed-effects	

Notes: *** *p* < 0.01, ** *p* < 0.05, * *p* < 0.1. Robust standard errors are shown in parentheses. Overall R^2^ is reported for the random-effects model and within R^2^ is reported for the fixed-effects models.
